# Acid-electrolyzed functional water-induces Interleukin-1α release from Intracellular Storage Sites in Oral Squamous Cell Carcinoma

**DOI:** 10.7150/ijms.53999

**Published:** 2021-02-18

**Authors:** Tomoko Takemoto, Ryo Kaetsu, Machiko Hanayama, Yuuichi Ishiyama, Masayuki Sadamura, Kensuke Nishio, Mariko Tsunoda, Masatake Asano, Mitsuru Motoyoshi

**Affiliations:** 1 Department of Orthodontics, Nihon University School of Dentistry, Tokyo, Japan.; 2 Division of Oral Structural and Functional Biology, Nihon University Graduate School of Dentistry, Tokyo, Japan.; 3 Division of Applied Oral Sciences, Nihon University Graduate School of Dentistry, Tokyo, Japan.; 4 Department of Complete Denture Prosthodontics, Nihon University School of Dentistry, Tokyo, Japan.; 5 Division of Advanced Dental Treatment, Dental Research Center, Nihon University School of Dentistry, Tokyo, Japan, 101-8310.; 6 Department of Pathology, Nihon University School of Dentistry, Tokyo, Japan.; 7 Division of Immunology and Pathobiology, Dental Research Center, Nihon University School of Dentistry, Tokyo, Japan.; 8 Division of Clinical Research, Dental Research Center, Nihon University School of Dentistry, Tokyo, Japan.

**Keywords:** acid-electrolyzed functional water, interleukin-1α, oral squamous cell carcinoma-derived cell line, alarmin

## Abstract

The aim of this study was to examine the acid-electrolyzed functional water (FW)-mediated cytokine release in an oral squamous cell carcinoma-derived cell line (OSCC) following treatment with FW. FW is generated by the electrolysis of a sodium chloride solution and accelerate the burn wound healing. To elucidate the underlying mechanisms, the cytokine/chemokine secretion profile of HSC3 cells was examined using a cytokine array. FW treatment significantly induced interleukin (IL)-1α secretion, which was confirmed by enzyme-linked immunosorbent assay. Subsequently, the HSC3 cells were pre-treated with cycloheximide (CHX) for 1 h prior to FW stimulation to determine whether the augmented IL-1α secretion was due to enhanced protein synthesis. CHX pre-treatment did not affect IL-1α secretion suggesting that the secreted IL-1α might have been derived from intracellular storage sites. The amount of IL-1α in the cell lysate of the FW-treated HSC3 cells was significantly lower than that of the non-treated cells. Immunofluorescence staining using a polyclonal antibody against full-length IL-1α revealed a drastic reduction in IL-1α inside the FW- treated cells. IL-1α is synthesized in its precursor form (pIL-1α) and cleaved to produce pro-piece and mature IL-1α (ppIL-1α and mIL-1α) inside the cells. In the present study, only pIL-1α was detected within the HSC3 cells in its resting state. However, FW stimulation resulted in the release of the 33 kDa and two other smaller forms (about 19 kDa) of the protein. These results indicates that FW treatment induces IL-1α secretion, a typical alarmin, from the intracellular storage in OSCC cells.

## Introduction

Acid-electrolyzed functional water (FW) is generated by electrolyzing low concentrations of saline 1, 2. Hypochlorous acid is the main component of FW at a concentration of approximately 10-60 ppm. The bactericidal effects of FW against drug-resistant bacteria and viruses are more potent than those of other chlorine-based disinfectants due to its acidic properties, despite the relatively lower levels of chloride 3. Moreover, FW was demonstrated to accelerate major burn healing by preventing burn-wound sepsis 4. Although the underlying mechanisms have not been elucidated, these beneficial effects of FW might be attributed to increased cytokine release. Previously, we demonstrated that the treatment of several cell lines with FW resulted in the release of various factors 5, 6, 7. Among them, the release of extracellular matrix metalloproteinase inducer (EMMPRIN) was significantly augmented in the human oral squamous cell carcinoma cell line (OSCC) HSC3 7. Augmented EMMPRIN secretion was not regulated at the transcriptional level and appeared to be released from intracellular storage sites. Based on these observations, EMMPRIN was proposed as a novel alarmin 7. Alarmins are molecules that are rapidly released from damaged cells 8 and function as early warning signals to activate the innate and adaptive immune systems.

In the present study, we examined the cytokine array experiment to identify the cytokines secreted in response to FW. The data demonstrated that IL-1α secretion was exclusively augmented by FW. IL-1α is a proinflammatory cytokine synthesized as a precursor (pIL-1α) inside the cells. pIL-1α is processed by calcium-dependent proteinase calpain within the cell to generate pro-piece IL-1α (ppIL-1α, N-terminal half of pIL-1α) and mature mIL-1α (C-terminal half of pIL-1α) 9. Due to the lack of a signal peptide, mIL-1α is secreted via the endoplasmic reticulum (ER)-Golgi-independent pathway 10. mIL-1α triggers various downstream inflammatory reactions through its cognate receptors. On the other hand, pIL-1α and ppIL-1α preferentially localize in the nucleus due to the nuclear localizing sequence (NLS) and regulate the expression of its target genes by activating transcription factors such as nuclear factor kappa beta (NF-κB) and activator protein -1 (AP-1) 11. Moreover, pIL-1α is reported to be released from cells undergoing necrosis 12, 13, 14. Intriguingly, both pIL-1α and mIL-1α were reported to induce IL-6 secretion from the human lung carcinoma cell line, A549 15. Taken together, these findings indicate the role of IL-1α as a typical alarmin and its activity has been demonstrated by the fundamental studies 13, 15, 16.

We demonstrate here that FW induces IL-1α secretion from the intracellular storage sites of OSCC. The molecular forms of secreted IL-1α is composed of two closely related 19 kDa forms in addition to 33 kDa pIL-1α. As only 33 kDa pIL-1α is released from resting cells, FW might enhance the cleavage of pIL-1α inside the cells.

## Materials and methods

### Reagents

FW, containing 20% Cl_2_ and 80% hypochlorous acid (pH, 2.2-2.7) was obtained using the Oxilyzer (Miura Denshi, Akita, Japan). Cycloheximide (CHX) was purchased from Sigma-Aldrich (Tokyo, Japan). The calpain inhibitor calpeptin was purchased from MedChemExpress (Monmouth Junction, NJ, USA).

### Cell culture and FW treatment

The HSC3 cells were maintained in RPMI1640 medium supplemented with 10% fetal calf serum (FCS), 50 μg/ml streptomycin, and 50 U/ml penicillin, in a 5% CO_2_ incubator. The cells (1 × 10^5^/24 well plate) were washed with phosphate-buffered saline (PBS) three times and treated with FW for 30 s. The reaction was stopped by adding an equal volume of the complete medium. After discarding the used medium, fresh medium was added to the cells, which were then allowed to incubate for 30, 60 and 180 min. For the CHX experiment, the cells were pre-treated with CHX (1 and 10 μM) for 1 h. Then, the cells were treated with or without FW, as described, and cultured. Antibody (Ab) inhibition was performed by treating the cells with or without anti- IL-1receptor type 1 (IL-1R1) Ab (100 ng/ml, Abcam) or a control Ab for 1 h prior to FW stimulation. For calpain inhibition, the cells were pre-treated with graded amount of calpeptin (0.1, 1, 10 μM) for 1 h prior to FW treatment. The lactate dehydrogenase (LDH) activity was measured with Lactate Dehydrogenase Activity Assay Kit (Sigma-Aldrich Japan, Tokyo, Japan).

### Cytokine array and ELISA

HSC3 cells were plated on to a 10-cm cell culture plate at a density of 1 × 10^6^ cells on the day before the experiment. The cells were treated with or without FW for 30 s. After washing, the cells were cultured for another 18 h. The culture supernatants were collected and subjected to cytokine array (R&D Systems, Minneapolis, MN, USA) according to the manufacturer's instruction. Briefly, the culture supernatants were centrifuged at 10,000 × g for 2 min and the supernatants were transferred to new tubes. Human cytokine array panel A membranes (R&D Systems) were incubated with FW-treated (Sup+) or -untreated (Sup-) HSC3 cell culture supernatants. After washing, the membranes were further incubated with a detection Ab cocktail for 1 h. Spot detection was performed with streptavidin-horseradish peroxidase incubation, followed by the use of the Enhanced Chemiluminescent (ECL) kit (GE Healthcare, Tokyo, Japan). The intensity of the spot was measured by the NIH Image software. The Sup+ and Sup- were subjected to IL-1α concentration measurements using the DuoSet enzyme-linked immunosorbent assay (ELISA) development system (R&D Systems). The absorbance was measured on a microplate reader (model 3550; Bio-Rad, Tokyo, Japan).

### Immunoprecipitation and Western blotting

The HSC3 cells were lysed with cell lysis buffer (50 mM Tris-HCl, pH 7.5, 150 mM NaCl, and 0.5% TritonX-100). The HSC3 cell lysate, Sup+, and Sup - were subjected to immunoprecipitation (IP) using rabbit anti-human IL-1α Ab (Abcam, Cambridge, UK) for 18 h at 4 °C. Ten μl of protein G-sepharose was added to the samples, which were rotated (Yamato Scientific, Tokyo, Japan) 50 rpm for 2 h. After rotation, the samples were washed with PBS or cell lysis buffer and loaded on to a 15 % SDS-PAGE gel. The separated samples were transferred to a membrane and subjected to Western blotting (WB), which was performed using rabbit anti-human mIL-1α Ab (Abcam) followed by horseradish peroxidase-conjugated goat anti-rabbit immunoglobulin (Ig)G (H+L) Ab. The bands were detected using an ECL kit (GE Healthcare).

### Immunofluorescence staining

The HSC3 cells were plated on to coverslips at a density of 5 × 10^4^/24 well/coverslip. After three washes with PBS, they were treated with or without FW for 30 s. The cells were further incubated with 10% FCS-RPMI for 3 h following which, they were fixed with 4% paraformaldehyde for 15 min. After washing with PBS, the permeabilization of the cells was performed by incubating them with 1% Triton X-100 solution (1% Triton X-100 in PBS) for 30 min. The cells were incubated with 1% BSA-PBS for 1 h to block the non-specific reaction; subsequently, they were incubated with rabbit anti-human IL-1α Ab (Abcam; ×100 dilution with 1% BSA-PBS) for another 18 h. After three wash with PBS, the cells were incubated with FITC-conjugated goat anti-rabbit IgG for 18 h. The cells were extensively washed with PBS and mounted on glass slides using Fluoroshield with DAPI (Gene Tex, California, USA). Images were taken using a fluorescence microscope (All-in-one Fluorescence Microscope, Keyence, Osaka, Japan).

### Statistical analysis

Statistically significant differences were determined by two-tailed Student's t-tests and Tukey's test. A p-value < 0.05 was considered significant. All data are plotted as means ± standard deviation (SD).

## Results

### Augmentation of IL-1α secretion following FW treatment

**Figure 1:**
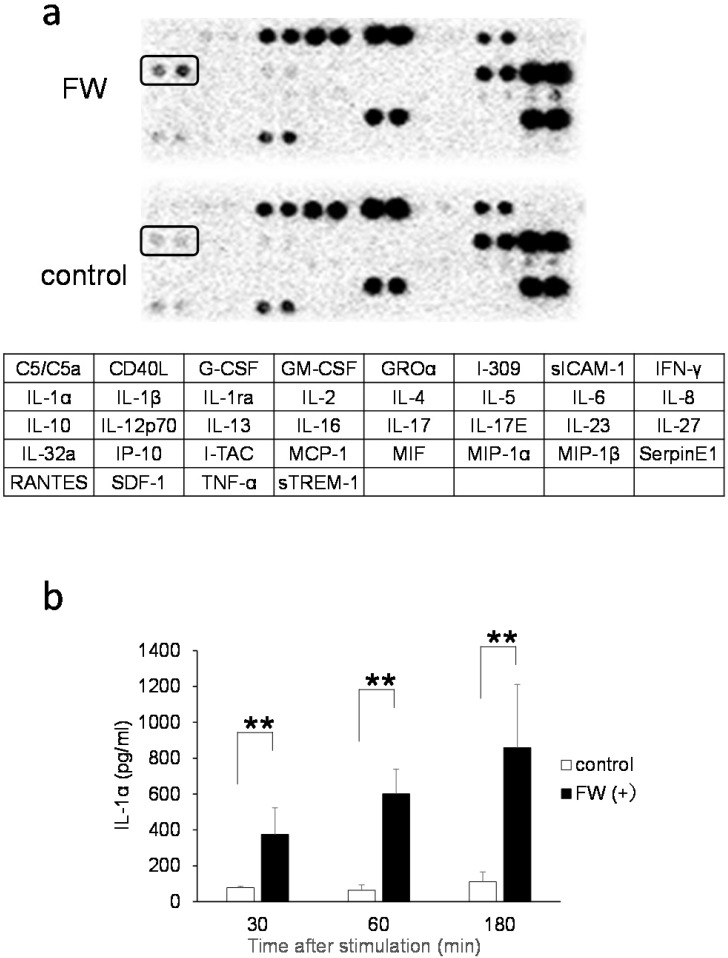
** IL-1α secretion by the HSC3 cells. a.** Cytokine array showing the amount of IL-1α secreted in cells treated with (upper panel) or without FW (lower panel) for 30 s. The IL-1α spots are indicated by the rectangles. The table below shows the spot position for each cytokine in the array. **b.** Bar graph showing enhanced secretion of IL-1α in the supernatants obtained from HSC3 cells treated with (black bars) or without FW (clear bars) for 30, 60, and 180 min via ELISA. The mean ± SD of at least five independent experiments are shown. **,*p* < 0.05.

A cytokine array experiment was conducted to examine the FW-mediated release of cytokines. The HSC3 cells were treated with or without FW and cultured for 18 h following which, the culture supernatants were collected and incubated using a factor-spotted membrane. Among the cytokines, IL-1α secretion was significantly augmented in the FW-treated culture supernatant; a 1.5-2-fold increase in the intensity of IL-1α spotting was observed in Sup+ (Fig. 1a, upper panel) when compared to that in Sup- (Fig. 1a, lower panel). To confirm the enhanced secretion of IL-1α, Sup + and Sup - were collected after 30, 60, and 180 min of FW treatment and subjected to ELISA. In Sup -, a gradual increase in the concentration of IL-1α from 77.5 ± 8.6 pg/mL at 30 min to 111.2 ± 54.5 pg/mL at 180 min was noted (Fig. 1b). In contrast, FW treatment markedly enhanced IL-1α secretion in Sup + from 375.0 ± 148.3pg/mL at 30 min to 601.3 ± 137.4pg/mL at 60 min and 859.0 ± 352.8 pg/mL at 180 min (Fig. 1b).

### New protein synthesis was not involved in the augmented secretion of IL-1α

**Figure 2:**
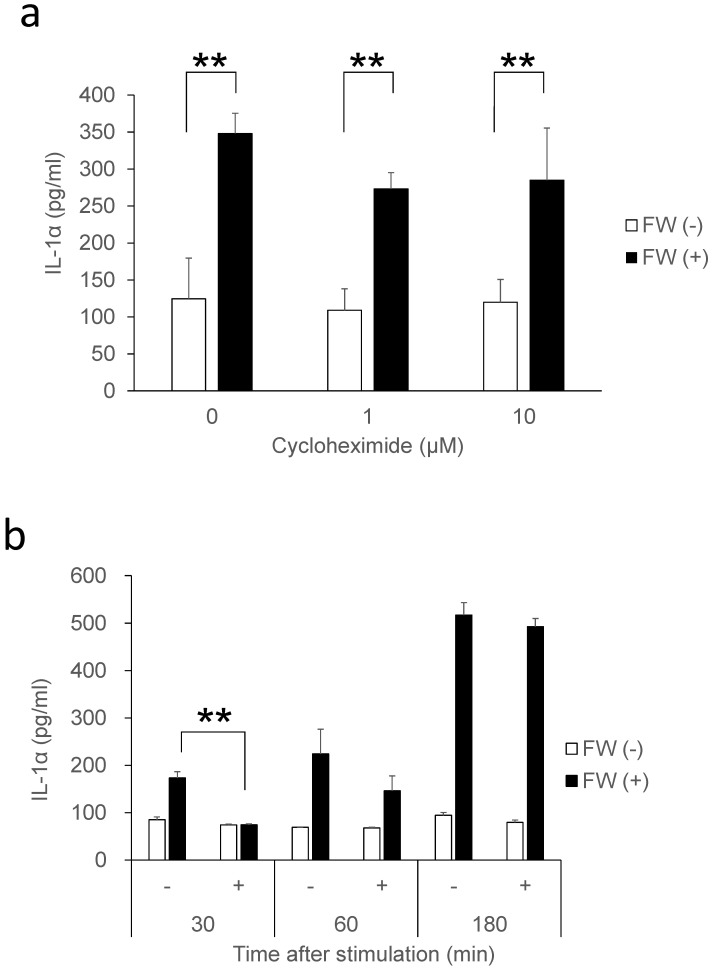
** IL-1α secretion in cells treated with CHX. a.** Bar graphs showing equivalent amounts of IL-1α secretion in the cells treated with 1 and 10 µM CHX when compared with the non-treated controls. **b.** Bar graph showing a time-dependent increase in IL-1α in the supernatants of HSC3 cells pre-treated with or without 1 µM CHX and stimulated with FW for 30 s. The culture supernatants were collected after 30, 60, and 180 min and subjected to IL-1α ELISA. The mean ± SD of at least three independent experiments are shown. **, *p* < 0.05.

**Figure 3:**
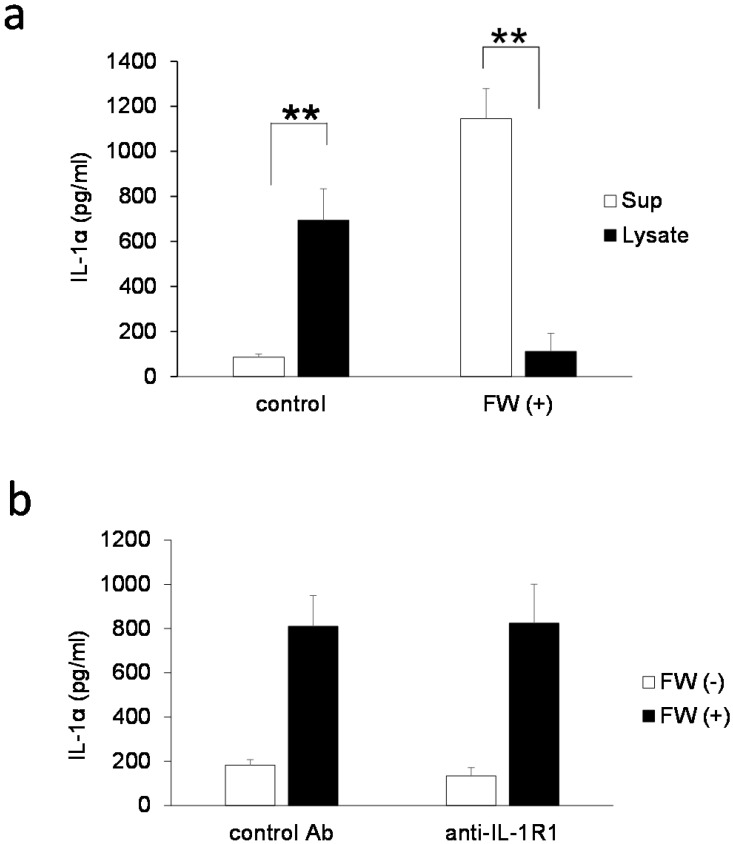
** IL-1α derived from intracellular storages. a.** Bar graph showing IL-1α levels in the cell lysates and supernatants of the HSC3 cells that were stimulated with (FW (+)) or without FW (control) for 30 s, and cultured for a further 18 h. The culture supernatants (Sup) and cell lysates (lysate) were collected and subjected to ELISA. **b.** HSC3 cells were pre-incubated with anti-IL-1R1 Ab or control Ab for 2 h and treated with FW for 30 s. The Sup was collected and subjected to ELISA. The mean ± SD of at least three independent experiments are shown. **, *p* < 0.05.

IL-1α is a typical alarmin, and the increased IL-1α in Sup + following FW treatment is thought to have been derived from intracellular stores. Consequently, the inhibition of new protein synthesis by CHX treatment might not affect the amount of IL-1α released in Sup +. To test this possibility, IL-1α concentration was measured after pre-incubation with or without CHX; pre-incubation with 1 μM (109 ± 29 pg/ml) or 10 μM (119 ± 30 pg/ml) of CHX did not affect the spontaneous secretion of IL-1α when compared with the CHX non-treated cells (0 μM: 124 ± 55 pg/ml). FW stimulation after CHX pre-treatment augmented IL-1α secretion to levels (CHX 1μM: 273 ± 21 pg/ml, 10 μM: 284 ± 70 pg/ml) equivalent to that in the CHX non-treated FW stimulated controls (347 ± 27 pg/ml) (Fig. 2a). The IL-1α secretion was further examined after 30, 60, and 180 min of FW stimulation with or without CHX pre-treatment; although a reduction in IL-1α secretion was observed after 30 min of FW stimulation following CHX pre-treatment, the levels were, subsequently, found to increase in a time-dependent manner (Fig. 2b). At 180 min, the amount of IL-1α secreted was similar to those in the CHX non-treated and FW-stimulated samples (Fig. 2b). These results indicated that the augmented IL-1α might be derived from intracellular storage sites. To confirm this assumption, the amount of IL-1α in the cell lysates was measured. The IL-1α concentration in cells without FW stimulation was 694.7 ± 139.2 pg/ml (Fig. 3a), whereas a drastic reduction to 111.6 ± 80.3 pg/ml was observed in cells treated with FW for 18 h (Fig. 3a). Furthermore, pre-treatment of HSC3 with or without anti-IL-1R1 Ab prior to FW stimulation did not affect the amount of IL-1α secreted from the cells (Fig. 3b), thus eliminating the possible contribution of autocrine IL-1α augmentation.

### Secretion of IL-1α from intracellular stores

**Figure 4:**
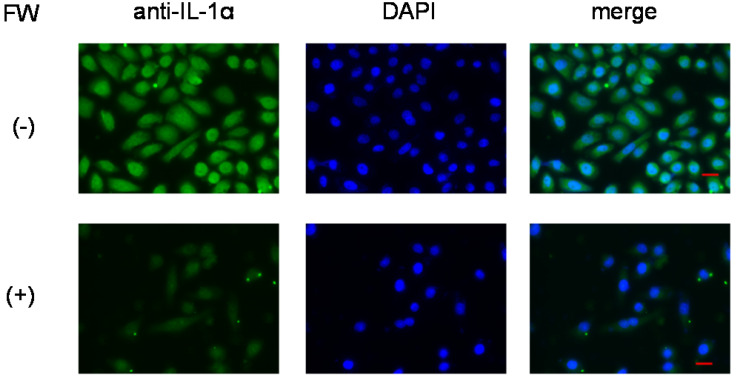
** Immunofluorescence staining of HSC3 cells.** After treatment with (lower panel, (+)) or without FW (upper panel, (-)), the cells were cultured for 3 h. Those without FW treatment showed strong fluorescence (-), mainly in the nucleus, and to a lesser extent in the cytoplasm. No fluorescence was observed in the FW-treated cells (+). The representative data of three independent experiments are shown.

To further confirm that IL-1α was released from intracellular stores, the HSC3 cells were subjected to immunofluorescence staining. Those without FW treatment showed strong fluorescence (Fig. 4, upper panel), mainly in the nucleus, and to a lesser extent in the cytoplasm. On the other hand, no fluorescence was observed in the FW-treated cells (Fig. 4, lower panel).

### IL-1α forms released in response to FW

**Figure 5:**
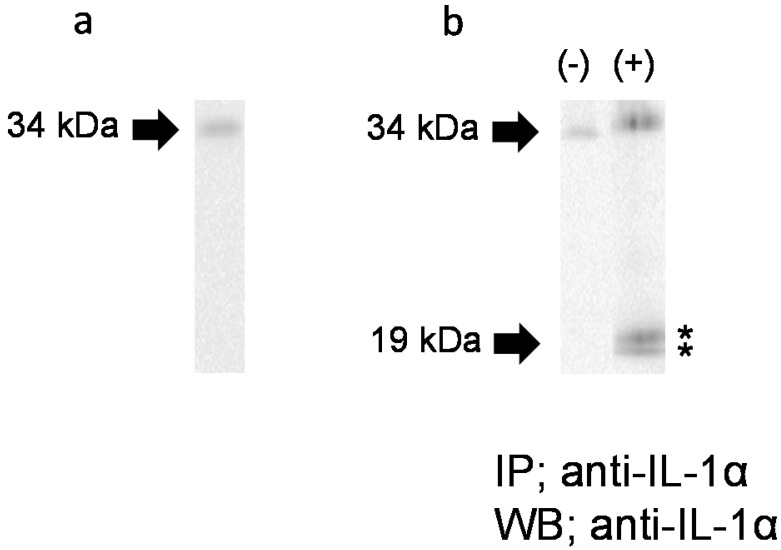
** Molecular forms of IL-1α secreted in response to FW. a.** A 33 kDa band was detected in the HSC3 cell lysate. **b.** The 33 kDa band was detected in FW non-stimulated HSC3 supernatant (left panel, (-)). In addition to the 33 kDa band, two bands (*) approximately19 kDa in size were detected in FW stimulated supernatant (+). The representative of five independent experiments are shown.

To further examine the IL-1α molecular form secreted in response to FW, we performed WB. As shown in Figure 5a, only a 33 kDa band was detected in the HSC3 cell lysate. When Sup- was subjected to IP-WB, only a single 33 kDa band was detected (Fig. 5b, left panel, (-)). On the other hand, two closely-loaded bands approximately 19 kDa in size were observed, in addition to the 33 kDa band, in FW-stimulated Sup+ (Fig. 5b, right panel, (+)). These results confirmed that the HSC3 cells secreted not only pIL-1α but also the 19 kDa form of IL-1α in response to FW.

### Calpain inhibition and lactate dehydrogenase release

**Figure 6:**
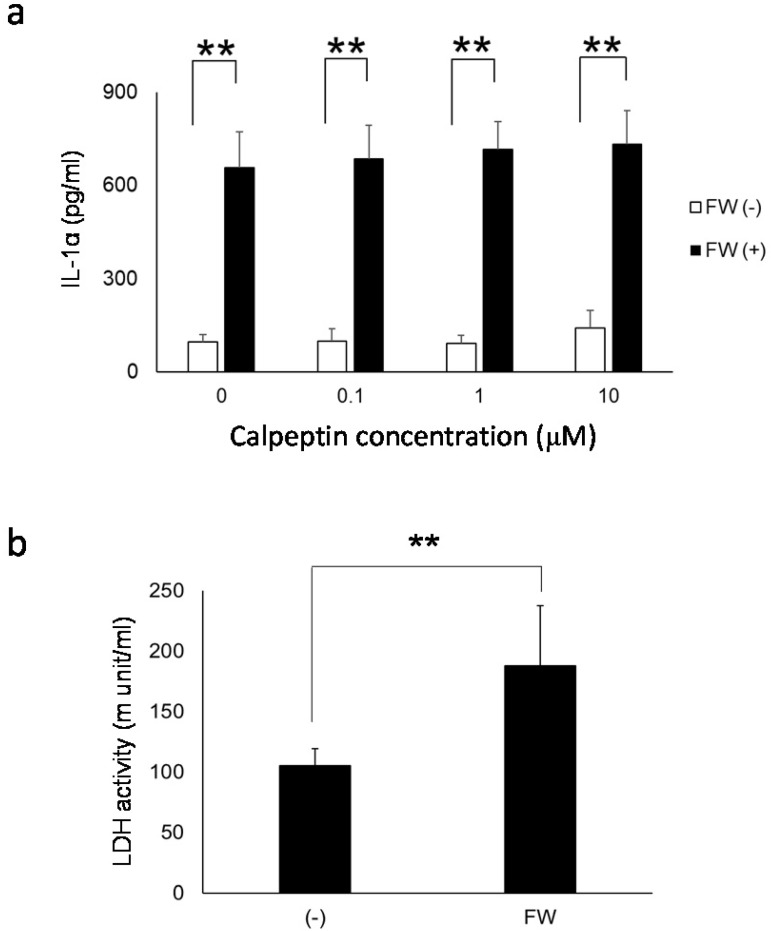
** a. Calpeptin pre-treatment did not affect the amount of IL-1α released into the supernatants.** HSC3 was pre-incubated with 0, 0.1, 1 and 10 μM of calpeptin for 1 h and further treated with FW for 30 sec. The supernatants were collected after 3 h of incubation and IL-1α concentration was measured with ELISA. Calpeptin pre-treatment did not affect IL-1α concentration in the culture supernatants. **b.** The extent of call damage. HSC3 was treated with or without FW for 30 sec and further cultured for 3 h. The LDH activity in the culture supernatants was measured with LDH activity assay kit. The LDH activity was augmented after 3 h of culture following the FW treatment. The mean ± SD of at least three independent experiments are shown. **, *p* < 0.05.

To further examine the contribution of pIL-1α processing enzyme calpain to IL-1α release from HSC3, the cells were pre-treated with or without calpeptin, a specific calpain inhibitor. As shown in Fig. 6a, IL-1α released in response to FW-stimulation was not affected by the calpeptin pre-treatment. Furthermore, the extent of cell damage after FW-treatment was examined by measuring the amount of LDH released in the culture supernatants. After 3 h of FW-treatment, the LDH released into the culture supernatants increased significantly (FW-treated; 188.2 ± 49.4 vs control; 105.6 ± 13.8) (Fig. 6b).

## Discussion

IL-1α and IL-1β share common molecular and functional properties as members of the IL-1 superfamily 17. Although they have only 27% homology at the amino acid level, they can bind to the same receptor (IL-1R1) due to similarities in the three-dimensional structure 18. They are initially synthesized as precursors (pIL-1α and pIL-1β) that enzymatically cleave to produce the mature forms (mIL-1α and mIL-1β) within the cell. Moreover, both molecules do not have a signal sequence and are secreted outside the cell via the unconventional ER-Golgi-independent pathway 10. Despite these similarities, FW treatment only induced the secretion of IL-1α (Fig. 1a), whereas the secretion of the other 34 cytokines was not augmented by FW.

FW-mediated IL-1α secretion was increased time-dependently (Fig. 1b). To exclude the possible contribution of autocrine IL-1α augmentation, the HSC3 cells were treated with anti-IL-1R1 Ab. As shown in Fig. 3b, the Ab did not affect the amount of IL-1α released by FW. Although FW-induced IL-1α secretion was slightly inhibited at the 30 min time point following CHX pre-treatment, it increased to levels similar to those seen in the CHX non-treated controls after 180 min of culture (Fig. 2b). Taken together, these results showed that the augmented IL-1α in Sup+ is derived from intracellular storage sites. Immunofluorescence staining revealed nuclear, and to a lesser extent, cytoplasmic localization of IL-1α in the HSC3 cells (Fig. 4). Due to the existence of NLS in the N-terminal region, pIL-1α preferentially localizes in the nucleus 19. In addition, pIL-1α has been shown to localize on the cell membrane through a lectin-like interactions 20 or myristoylation 21. More importantly, nuclear localization and release of IL-1α is affected by the acetylation of NLS 22, 23. FW treatment drastically reduced the fluorescence intensity in all these compartments (Fig. 4) and the mechanisms underlying should be examined by further experiments.

IL-1α is detected in a variety of non-myeloid cells such as keratinocytes, endothelial cells, and fibroblasts 24. The enzymes that cleave pIL-1α into ppIL-1α and mIL-1α are calpain 25, 26, granzyme B (GzmB), elastase, mast cell chimase and thrombin 27, 28, 29. Due to the abundance of calpain inhibitors, non-myeloid cells cannot produce mIL-1α inside the cells 26. Consistent with these reports, only the 33 kDa pIL-1α was detected in the HSC3 cells in this study (Fig. 5a).

The IP-WB experiment was performed to reveal the form of IL-1α released in response to FW. Surprisingly, two bands of about 19 kDa were detected in Sup+, in addition to the 33 kDa band (Fig. 5b). Theoretically, the Ab used in IP can recognize pIL-1α, ppIL-1α, and mIL-1α because it is obtained by immunizing pIL-1α. Hence, the two additional bands detected in Sup+ might correspond to mIL-1α and ppIL-1α. Previously, we developed a polyclonal Ab that recognizes ppIL-1α by immunizing rats with histidine-tagged ppIL-1α 30. However, both bands could not be recognized by this Ab (data not shown) due to its low sensitivity. Nonetheless, additional studies are required to determine the nature of these two bands.

Both pIL-1α and mIL-1α are biologically active in the extracellular milieu and can bind to IL-1R1 15. Furthermore, as shown in this study, pIL-1α was secreted by HSC3 in response to FW. These facts suggested that the secreted pIL-1α could interact with its cleaving enzyme, such as GzmB. GzmB is secreted into the extracellular space, and its level in the serum is significantly increased in patients with rheumatoid arthritis 31. Thus, the cleavage of pIL-1α could result in the generation of pp and mIL-1α, extracellularly. Although the extracellular function of ppIL-1α has never been examined, this phenomenon could increase the immunological role of IL-1α. The mechanisms involved in the release of 19 kDa IL-1α in the Sup+ are unknown. The treatment of macrophages with calcium ionophore has been shown to activate calpain, a Ca^2+^-dependent enzyme 32, resulting in the cleavage of pIL-1α 33. FW might induce the influx or release of calcium from the intracellular storage sites, such as the mitochondria, and generate pp and mIL-1α. To further examine the contribution of calpain on 19 kDa IL-1α release, HSC3 was pre-incubated with calpain inhibitor calpeptin prior to FW stimulation. However, calpeptin did not affect the amount of IL-1α released in response to FW treatment (Fig. 6a). As IL-1α is released not only from dying cells (8. 9) but also from live cells 22, 23, 34, 35, 36, 37, we further attempted to examine the extent of cell damage by measuring LDH activity. As the LDH activity was significantly augmented in FW-treated cells (Fig. 6b), the cells are expected to be damaged to some extent.

FW treatment augmented the release of IL-1α from HSC3. The augmentation was not accompanied with increased IL-1α mRNA transcription. Anti-IL-1R1 Ab blocking did not affect IL-1α. Moreover, inhibition of new protein synthesis by CHX had no effects on IL-1α release. Taken all these results together, we speculated here that IL-1α released in response to FW treatment is passively released by the damaged cells. Recently, IL-1α was demonstrated to detect DNA damage in living cells and actively released to the outside of the cells to evoke the inflammatory reactions 38. With these functions, IL-1α is denoted as “Stressorin”. FW-treatment totally abolished the cellular IL-1α by immunofluorescence staining (Fig. 4) and we could not trace the path of IL-1α movement inside the cells. For these reasons, we could not detect the stressorin function of IL-1α in the present study. However, the properties and mechanisms involved in the generation of the two 19 kDa bands should be examined in future experiments.

## Conclusion

In the present study, FW treatment was shown to induce IL-1α secretion in HSC3 cells. The secreted form contained 33 kDa precursor form and two 19 kDa forms; hence, FW might be able to enhance the cleavage of pIL-1α. Elucidation of the mechanisms underlying this phenomenon should extend our understanding of the properties of IL-1α and enlighten us about the benefits of using FW.
